# Oleanolic Acid Controls Allergic and Inflammatory Responses in Experimental Allergic Conjunctivitis

**DOI:** 10.1371/journal.pone.0091282

**Published:** 2014-04-03

**Authors:** Claudia Córdova, Beatriz Gutiérrez, Carmen Martínez-García, Rubén Martín, Patricia Gallego-Muñoz, Marita Hernández, María L. Nieto

**Affiliations:** 1 Instituto de Biología y Genética Molecular, Consejo Superior de Investigaciones Científicas-Universidad de Valladolid, Valladolid, Spain; 2 Departamento de Biología Celular, Histología y Farmacología, Universidad de Valladolid, Valladolid, Spain; 3 Instituto de Ciencias del Corazón. Hospital Clínico Universitario, Valladolid, Spain; Macau University of Science and Technology, Macau

## Abstract

Pollen is the most common aeroallergen to cause seasonal conjunctivitis. The result of allergen exposure is a strong Th2-mediated response along with conjunctival mast cell degranulation and eosinophilic infiltration. Oleanolic acid (OA) is natural a triterpene that displays strong anti-inflammatory and immunomodulatory properties being an active anti-allergic molecule on hypersensitivity reaction models. However, its effect on inflammatory ocular disorders including conjunctivits, has not yet been addressed. Hence, using a Ragweed pollen (RWP)-specific allergic conjunctivitis (EAC) mouse model we study here whether OA could modify responses associated to allergic processes. We found that OA treatment restricted mast cell degranulation and infiltration of eosinophils in conjunctival tissue and decreased allergen-specific Igs levels in EAC mice. Th2-type cytokines, secreted phospholipase A_2_ type-IIA (sPLA_2_-IIA), and chemokines levels were also significantly diminished in the conjunctiva and serum of OA-treated EAC mice. Moreover, OA treatment also suppressed RWP-specific T-cell proliferation. In vitro studies, on relevant cells of the allergic process, revealed that OA reduced the proliferative and migratory response, as well as the synthesis of proinflammatory mediators on EoL-1 eosinophils and RBL-2H3 mast cells exposed to allergic and/or crucial inflammatory stimuli such as RWP, sPLA_2_-IIA or eotaxin. Taken together, these findings demonstrate the beneficial activity of OA in ocular allergic processes and may provide a new intervention strategy and potential therapy for allergic diseases.

## Introduction

Allergic conjunctivitis (AC) is one of the most common ocular surface diseases. The disease encompasses a variety of pathological conditions, and based on immunopathological mechanisms, it can be subdivided into seasonal and perennial allergic conjunctivitis [1]. AC is an abnormal immune-hypersensitivity response to allergens mainly pollen, animal dander and house dust mites, although some food substances may also trigger it. They have common immunopathogenic mechanisms characterized by IgE-mediated mast cell degranulation and/or T-lymphocyte-mediated immune response. Allergen-specific Th2-type lymphocytes play important roles in the immunopathophysiology of allergic disorders because of their ability to produce IL-4, IL-5 and IL-13, which are involved in IgE production and eosinophil activation [2].

Medications for AC include drug treatment (anti-histamines, mast cell stabilizers, corticosteroids, non-steroidal anti-inflammatories, immunomodulatories), and allergen-specific immunotherapy. However, its effectiveness can be endangered by adherence problems related to factors, such as discomfort associated with treatment administration, complexity of administration guidelines, perception of a lack of efficacy at treatment initiation, and/or adverse effects. Therefore, the development of new therapeutic strategies will be a valuable tool to achieve better control of the disease and, thus, will improve healthcare outcome for patients with allergic conjunctivitis.

Because alternative treatments are needed, plants and other natural materials may prove to be valuable sources of useful new anti-allergic agents [3]. Research groups have conducted clinical trials with remedies from complementary and alternative medicine [4]–[7]. Plant formulations have demonstrated, in general, to be safe, revealing additional effects along with Western medicines such as synergism and modulation of the immune system. Triterpenes, including oleanolic acid (OA) are compounds that widely exist in the human diet, medicinal herbs and plants. OA has been identified in more than 120 plant species, including *olea europaea*, a plant that has gained more and more interest because of its multiple bioactive components [8]. Among the numerous beneficial effects, OA has been shown to have cardioprotective, antihypertensive, antiatherosclerotic, antihyperlipidemic and antioxidant activities, among other, as well as anti-inflammatory and immunomodulatory properties, being active on hypersensitivity reactions, such as the delayed-type hypersensitivity reaction, or allergic asthma, and in Th1-mediated diseases, including experimental colitis and multiple sclerosis [9]–[16]. However, the effectiveness of OA in the treatment of ocular diseases such as allergic conjunctivitis is still unknown.

The IgE-mediated conjunctival allergic reaction can be reproduced easily in mice by using RWP as antigen [17]. The result is an early reaction followed by a predominant infiltration of eosinophilic inflammatory cells, which are the hallmark of allergic disease, and mast cell activation. This experimental allergic conjunctivitis (EAC) model mimics the pathological symptoms of AC in human, being a valuable tool to understand the pathogenesis of the disease, as well as for new drugs evaluation. The aim of this study was, therefore, to evaluate the effectiveness of OA using this well-characterized EAC model induced by RWP in BALB/c mice.

## Materials and Methods

### Disease Induction and Treatment

Animal care and experimental protocols were reviewed and approved by the Animal Ethics Committee of the University of Valladolid and complied the standards in the ARVO Statement. Mice (Charles River Laboratories, Barcelona, Spain) were housed in the animal facilities of the University of Valladolid and provided food and water ad libitum.

#### Immunization

EAC was induced in 6- to 7-week-old females BALB/c mice by intraperitoneal (i.p.) sensitization with 200 μl from a mixture of 50 μg RWP (Polyscience, Warrington, PA) in 0.25 ml Imject Alum (Thermo Scienctific, Rockford, IL). Then, on day 10, mice were topically instilled into each eye with 1.25 mg of RWP in powder form [3], [18]. Twenty-four hours later, eyes, blood and spleens were collected.

#### Treatment

**Figure 1:**
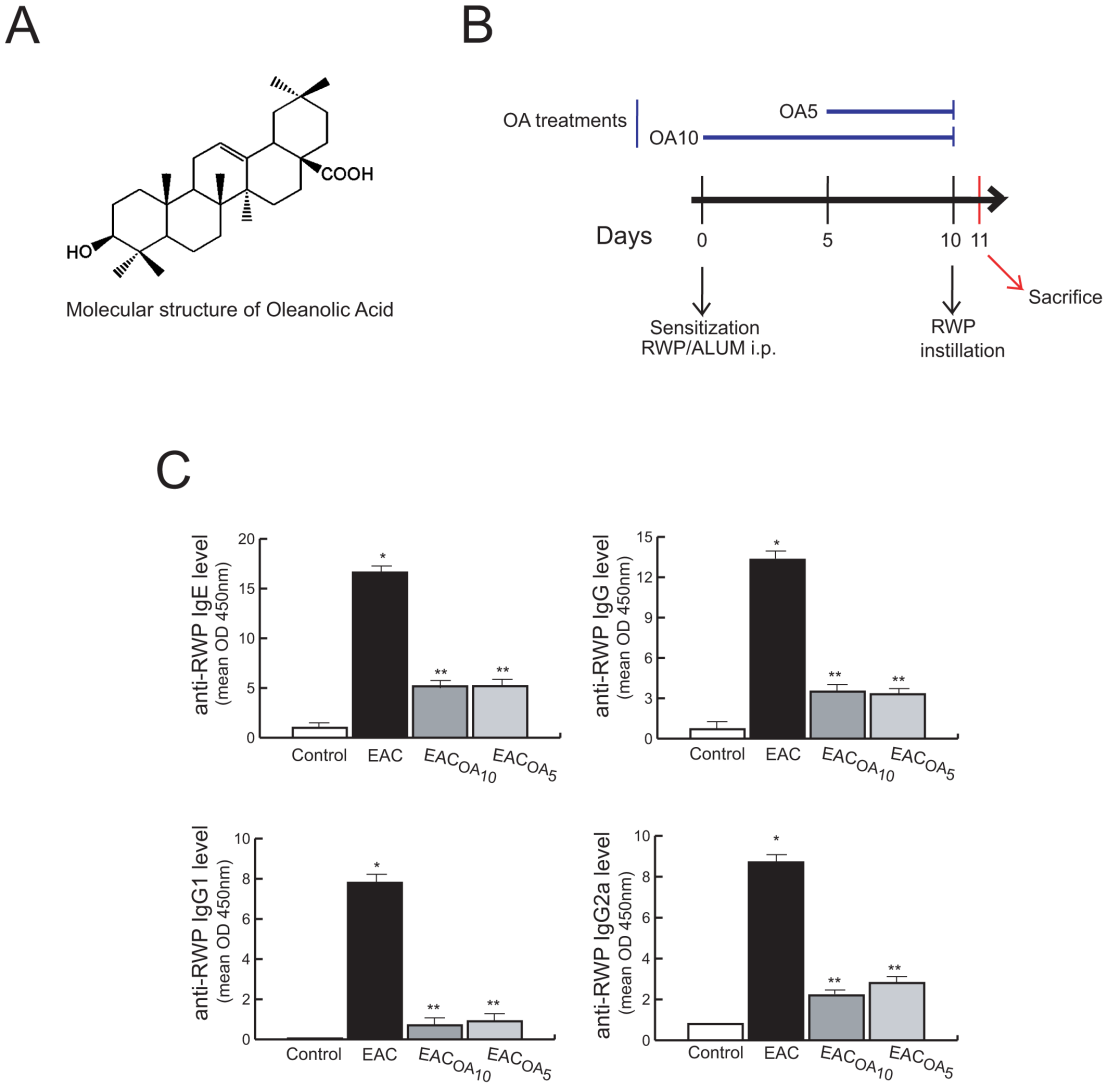
Reduction of circulating anti-RWP antibodies in OA-treated mice. Molecular structure of OA (A). Experimental protocol (B). BALB/c mice were sensitized on day 0 via i.p. injection with RWP emulsified in aluminum hydroxide. 10 days latter, individual mice were challenged through ocular instillation with RWP. OA was administered i.p. at the sensitization day (OA_10_) or 5 days after sensitization (OA_5_). Animals were sacrificed 24 h after the challenge. (C) Titers of RWP-specific immunoglobulins in serum samples. *P<0.001 versus control, and **P<0.001 versus untreated EAC-mice.

Oleanolic acid (Extrasynthese, Genay Cedex, France) was dissolved in 2% w/v DMSO. Then, this stock solution was 10 times diluted to a working concentration of 2 mg/ml (0.2%, w/v DMSO final concentration), using a sterile saline solution (pre-warmed to 37°C) with mild shaking. Always, OA working solution was prepared fresh the day of the injection and sterile-filtered through a 0.22 μm filter. OA (50 mg/kg body weight/day) was administered i.p. once a day from sensitization day (OA_10_) or 5 days after sensitization (OA_5_) as shown in Figure 1B (n = 10 per group). The molecular structure of OA is shown in Fig. 1A (OA molecular formula: C_30_H_48_O_3_, molecular weight: 456.70).

### Evaluation of Cell Infiltration

Eyes including eyelids and conjunctiva were exenterated and fixed in 4% paraformaldehyde for 24 h. Then, they were cut into cross sections 3-μm thick and stained with toluidine blue and hematoxylin-eosine for detection of mast cells and eosinophils, respectively. In each section, infiltrating cells in the lamina propria mucosae of the palpebral and bulbar conjunctivas were counted by two masked observers. Palpebral zone: connective tissue between the epithelium and the Meibomio gland in the centre of the lid. Bulbar conjunctiva: near the cornea in reflection zone that form the conjunctival sac. In each slide there were two non-consecutive sections and three slides per eye and per stain were counted. An Olympus B–H (Olympus optical Co. LTD, Tokio, Japan) microscope and a micrometer ocular grid were used to evaluate the slides (magnification x400). Data are presented as mean ± SD per mm^2^.

### Cytokines, Chemokines and RWP-specific Igs Quantitation

Serum was aliquoted and stored at −80°C. Immunoplates (Nalge Nunc International, Naperville, IL) were coated with RWP (5 mg/ml) overnight at 4°C. After blocking with 1% BSA-PBS for 2 h, serum samples diluted 1∶60 were added and incubated for 3 h. After washing, plates were incubated for 1 h with horseradish peroxidase-conjugated rat anti-mouse IgE, IgG, IgG1 or IgG2a antibodies (1∶2000) from Serotec (Sigma-Aldrich, St Louis, MO). Color reaction was developed with 3,3′,5,5′-tetramethyl-benzidine and stopped with 0.1N HCl. Data are expressed as mean optical density (OD) at 450 nm.

For cytokine and chemokine quantification - IL-10, IL-13, IL-33, monocyte chemoattractant protein-1 (MCP-1) and eotaxin - cell culture medium, serum and conjunctival tissue were analyzed by ELISA according to the manufacturer’s protocols (eBioscience, San Diego, CA). Secreted phospholipase A_2_-typeIIA (sPLA_2_-IIA) levels were determined by commercial ELISA (USCNK Life Science Inc, Houston, TX). Conjunctival tissue was homogenized by using a tissue homogenizer (Cole-Parmer Instrument, Vernon Hills, IL) in an ice bath in 0.5 ml ice-cold PBS supplemented with 0.4 M NaCl, 0.05% Tween-20 and a protease inhibitor cocktail: 20 mg/ml of leupeptin, 20 KI units of aprotinin, 0.1 mM phenylmethylsulphonyl fluoride, and centrifuged for 10 min at 4°C. Supernatant were stored at −80°C until cytokine assays were performed. Total protein was assayed using the Bradford method.

### Ex vivo Lymphoid Cell Culture and Analysis of Proliferation and Cytokine Production

To prepared single cell suspension, spleens were pressed though a wire mesh and then washed with ice-cold PBS. Red blood cells-depleted splenocytes (10×10^6^ cells/ml) were cultured in triplicate in 96-well plates, and stimulated with medium alone or with 50 μg/ml of RWP in presence or absence of different doses of OA. After 24 h, supernatants were harvested and concentrations of IL-33, IL-13 and MCP-1 were measured. Cells were prepared for proliferation analyses using the Promega kit, Cell Titer 96^R^Aqueous One Solution Cell Proliferation Assay (Promega Corporation, Madison, WI), according to the manufacturer’s recommendations. Formazan product formation was determined by measuring the absorbance at 490 nm. Results were expressed as OD values, as an assessment of the number of metabolically active cells.

### In vitro Studies

#### Cell culture

The rat basophilic leukemia (RBL) mast cell line RBL-2H3 (kindly provided by Dr. Ruiz; Hospital Universitario Puerta del Mar, Facultad de Medicina, Universidad de Cádiz, Spain) was grown in EMEM supplemented with 15% heat-inactivated FCS, 0.1 mM nonessential amino acids, 50 U/ml penicillin, and 50 μg/ml streptomycin [19].

The human eosinophilic cell line EoL-1 (kindly provided by Dr. Wicklein, University Medical-Center Hamburg-Eppendorf, Hamburg, Germany) was grown in RPMI 1640 medium, supplemented with 10% heat-inactivated FCS, 2 mM L-glutamine, 100 U/ml penicillin, and 100 μg/ml streptomycin [20], [21].

Both cell lines were cultured in standard conditions (37°C and 5% CO2).

#### Proliferation assay

The proliferative activity of EoL-1 and RBL-2H3 cells was quantified using the Promega kit, Cell Titer 96^R^Aqueous One Solution Cell Proliferation Assay, as described for the ex vivo proliferation assay. In brief, cells (1×10^5^ cells/ml) were serum-starved for 24 h, incubated as indicated and measured. Results were expressed as OD values.

#### Chemotaxis assay

Cell migration was assayed using 24-well Transwell chambers with 8 μm-pore polycarbonate filters. Lower wells were filled with medium containing eotaxin, sPLA_2_-IIA or vehicle, as indicated. Cells (1×10^6^ cells/ml) were added to the upper chamber. When indicated, cells were treated with sPLA_2_-IIA, RWP or OA for 20 min at 37°C before seeding. Migration was allowed for 3–4 h at 37°C in 5% CO_2_. Migrated RHL-2H3 cells were fixed with 4% paraformaldehyde and stained with 0.1% Crystal Violet in 20% methanol-PBS for 3 minutes. The stain was solubilized and absorbance measured at 590 nm. Migrated EoL-1 cells were quantified by flow cytometry analysis. Chemotactic index was calculated from the number of cells migrating towards chemoatractant/number of cells migrating towards vehicle control.

#### Flow cytometry of intracellular major basic protein (MBP)

EoL-1 cells were treated with butyric acid, or the stimuli eotaxin, RWP, sPLA_2_-IIA or combinations for 7 days. Then, cells were washed with 0.5% BSA-PBS, fixed with 4% paraformaldehyde for 30 min and permeabilized with 0.2% Triton-X 100 in PBS for 10 min. Non-specific antibody binding was blocked by incubating cells with normal mouse IgG for 30 min. Then, cells were stained with an anti-MBP antibody (1∶200) for 2 h. After washing, they were incubated with FITC-labeled goat anti-mouse IgG for 1 h at 4°C. Baseline fluorescence values were obtained by incubation with isotype mouse IgG antibody. After washing, the stained cells were analyzed using a Gallios Flow cytometer (Beckman Coulter), and acquisition and analysis were performed using CellQuest software. The data are presented as an average of mean intensity fluorescence (MIF).

### Statistical Analysis

Statistical significance between groups was examined by one-way ANOVA using the GraphPad Prism Version 4 software (San Diego, CA). A post hoc analysis was made by the Bonferroni’s multiple comparison test. P<0.05 was considered statistically significant.

## Results

### OA Treatment Inhibits RWP-specific Antibody Production in EAC Mice

To investigate whether OA treatment modulates adaptive immune response to conjunctival allergens, as well as the influence of the pre-treatment period in its effectiveness, EAC mice were treated with OA from sensitization day (OA_10_) or 5 days after sensitization (OA_5_) as shown in Figure 1B. The severity of EAC was elucidated by assessing RWP-specific IgE antibody levels in serum. The combination of systemic sensitization and local boosting with RWP resulted in significantly higher levels of Ag-specific IgE antibody secretion (Fig. 1C) compared with those in normal mice. However, the administration of OA, both under OA_10_ and OA_5_ protocols, led to a dramatic and similar reduction of Ag-specific IgE antibody levels (Fig. 1C). To address overall immunosuppression by OA, we also assessed levels of RWP-specific IgG, IgG1 and IgG2a antibodies in serum, finding similar results to those obtained for RWP-specific IgE antibodies. Taken together, these data suggest that oleanolic acid suppressed both allergen specific IgE production and overall immune responses.

### OA Treatment Attenuates Cellular Infiltration into Conjunctiva in EAC Mice

**Figure 2:**
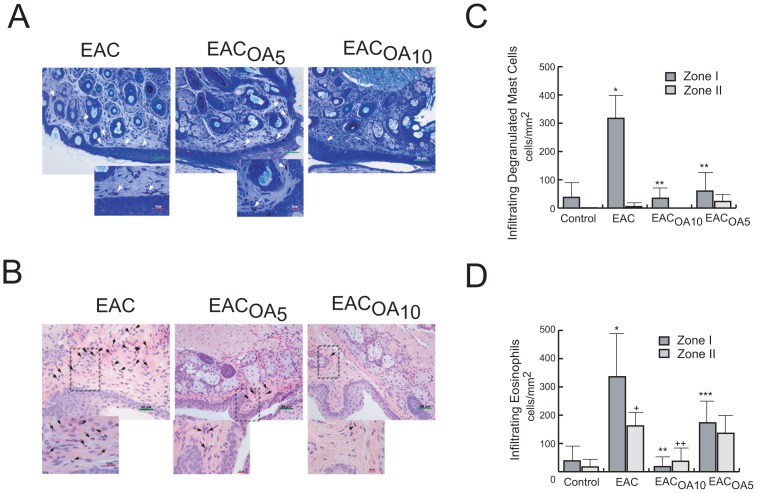
OA administration prevents development of allergic conjunctival inflammation. Light micrograph of conjunctival sections in mice from allergic untreated, OA_5_-treated and OA_10_-treated groups. Representative toluidine (A) and H-E (B) stained sections showed infiltration in palpebral conjunctiva of mast cells and eosinophils, respectively. (C, D) Number of degranulated mast cells/mm^2^ and eosinophils/mm^2^, respectively. In images, green bars = 50 μm. In inserts, red bars = 10 μm. * and **P<0.01 (n = 10, three independent experiments). Zone I: conjunctiva palpebral. Zone II: conjunctiva bulbar. * and^ +^P<0.001 versus control zone I and zone II, respectively; ** and^ ++^P<0.001 versus untreated EAC-mice zone I and zone II, respectively; and ***P<0.01 versus untreated EAC-mice zone I.

Histological findings demonstrated by toluidine blue staining the presence of numerous mast cells infiltrating lamina propria and stroma of conjunctiva in EAC mice, being most of them degranulated mast cells (Fig. 2A and C). In contrast, in the OA-treated EAC groups infiltrating mast cells were mainly granulated and the number of degranulated mast cells was significant lower, compared to untreated ones.

In the same way, hematoxylin-eosine staining revealed a significant infiltration of eosinophils in the conjunctiva of RWP-challenged mice 24 h after systemic priming (Fig. 2B and D). Interestingly, the number of eosinophils infiltrating the conjunctiva after allergen challenge was minimal in mice treated with OA.

Mast cell and eosinophil counts were not significantly different between EAC mice injected with either oleanolic acid regimens (OA_10_ or OA_5_).

### OA Treatment Reduces the Release of EAC-associated Cytokines and Chemokines

**Figure 3:**
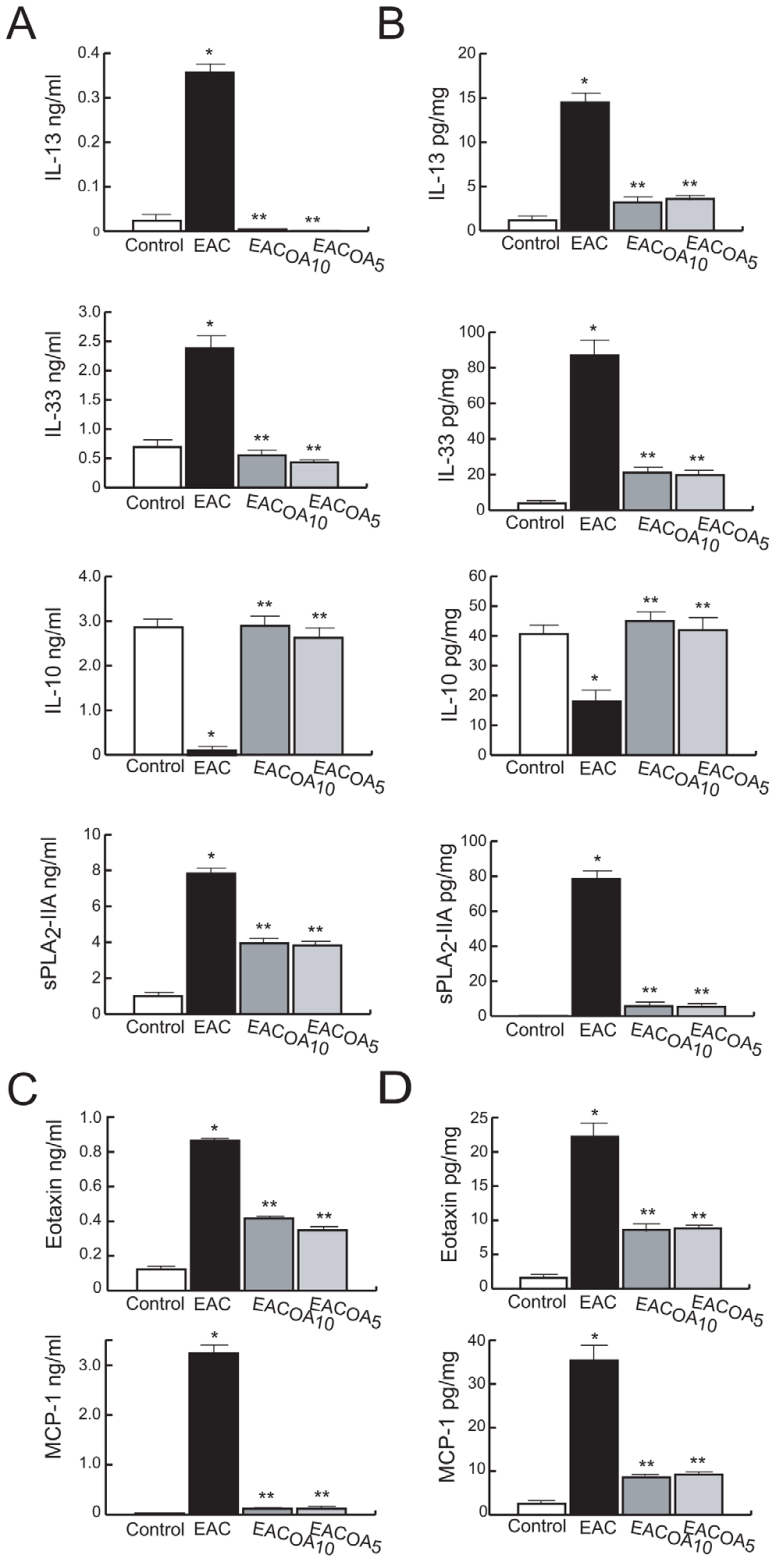
Effect of OA on cytokine and chemokine levels in EAC mice. The cytokines IL-13, IL-33, IL-10 and sPLA_2_-IIA (A,B), as well as the chemokines eotaxin and MCP-1 (C,D) were quantified in serum samples (A,C) and ocular conjunctiva extracts (B,D) from mice of the indicated groups 24 h after challenge. Results were expressed as the mean ± SD (n = 7/group). *P<0.001 vs control, and **P<0.001 vs untreated-EAC mice.

Th2-associated cytokines and chemokines are typically secreted in allergic reactions. To evaluate the effect of OA on cytokine secretion, levels of IL-13 and IL-33 were investigated in serum and conjunctival tissue by commercial ELISAs. As shown in Figure 3 A and B, IL-33 and IL-13 were remarkably up-regulated in the allergic state and OA treatment diminished their expression levels.

We also evaluated the presence of IL-10, a known suppressive cytokine of T-cell proliferation and cytokine production, in normal, allergic and OA-treated allergic mice. We found that IL-10 levels in serum and tissue were significantly diminished in the allergic state, but were up-regulated reaching values of the normal state, in OA-treated EAC mice.

Production and build up of sPLA_2_-IIA, an acute-phase reactant associated with a number of inflammatory, autoimmune, and allergic diseases, was also evaluated [22]. sPLA_2_-IIA was augmented in serum and conjunctivas of mice from the untreated-EAC group, compared with the healthy-control group (Fig. 3 A and B). However, and according to Th2-type cytokines data, we found that this inflammatory protein was significantly lower in OA-treated EAC mice, at both systemic and local level.

Moreover, we also measured the concentration of two chemokines: eotaxin, given its critical role in eosinophil recruitment, Th2-related cytokines production and mast cell priming; and MCP-1, which is essential for mast cell-mediated acute inflammatory responses [23]. Again, we observed that OA treatment suppressed the high levels of eotaxin and MCP-1 found in sera and conjunctivas of allergic mice (Fig. 3 C and D).

There was no difference in regulating these conjunctivitis-associated markers between the two treatment protocols: EAC_OA5_ and EAC_OA10_.

### OA Treatment Regulates T Cell Responses in EAC

Next, to investigate whether OA treatment inhibits EAC by modifying/affecting systemic immune responses, we evaluated the function of splenocytes harvested from mice of the four experimental groups.

**Figure 4:**
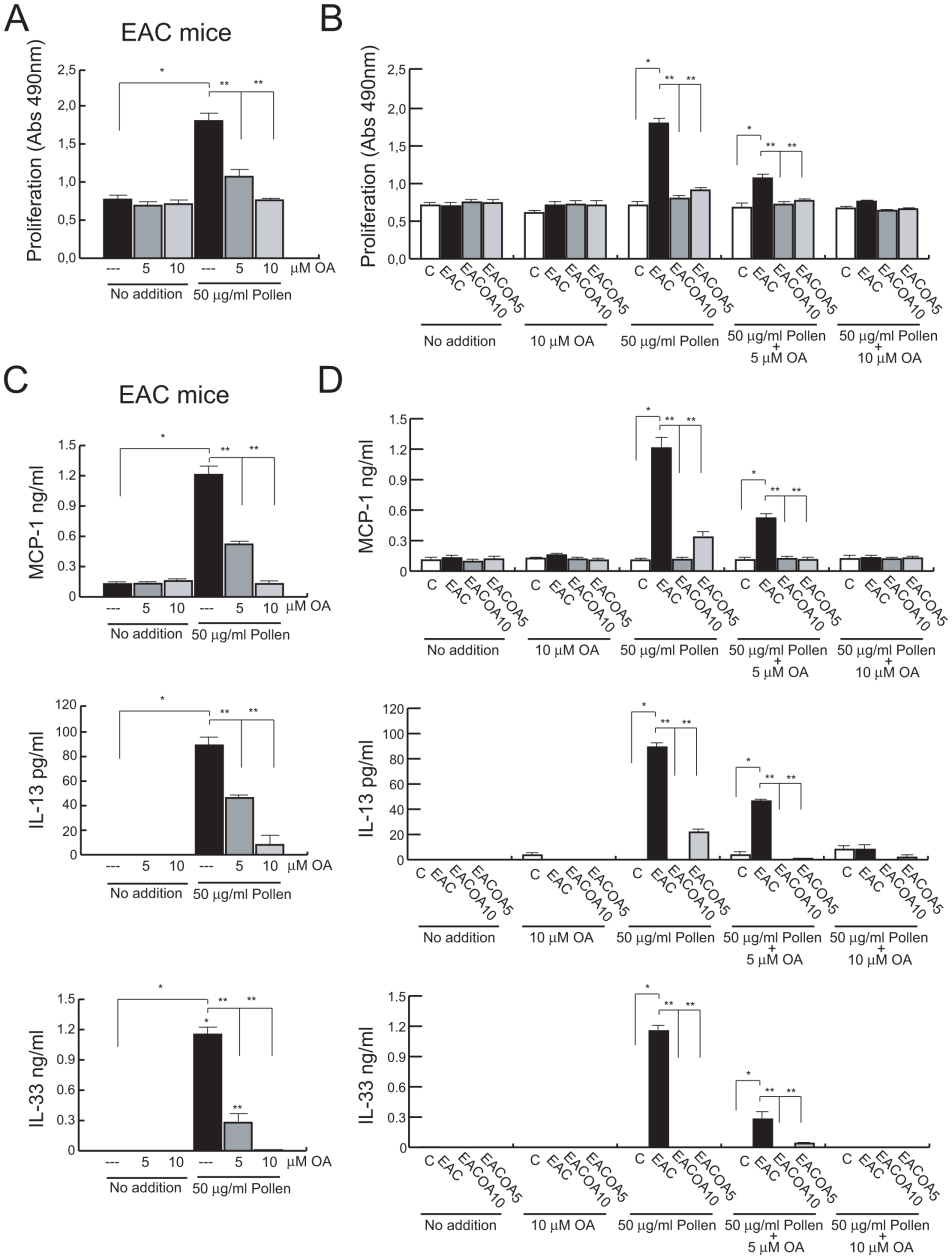
Effect of OA on splenocyte functions. RWP-specific proliferative responses of splenic cells from untreated-EAC mice (A), and from mice of the indicated groups (B). Cells were treated for 24 h with 50 μg/ml of RWP in presence or absence of the indicated doses of OA, and proliferation was measured. Each bar represents the mean ± SD. (C and D) Quantification of IL-13, IL-33, and MCP-1 in supernatants from (A) and (B). Bars represents means ± SD. (n = 5). * and **P<0.001.

Spleen cells were assessed for antigen-specific proliferation and cytokine secretion after *in vitro* stimulation with RWP in the presence or absence of different doses of OA. RWP-specific proliferative response, as well as IL-13, IL-33 and MCP-1 production were significantly increased in spleen cells from mice with EAC, and addition of OA markedly suppressed, in a dose-dependent manner, these ex-vivo responses to the allergen (Fig. 4, A and C). In contrast, splenocytes isolated from healthy-control mice did not respond to in vitro RWP challenge, and no major responses were detected in spleen cells from EAC_OA10_ and EAC_OA5_ mice (Fig. 4, B and D).

### OA Inhibits in vitro Functional Activation of Allergy-related Cells

We next investigated whether the anti-inflammatory effect found *in vivo*, in OA-treated EAC mice, comprises also direct *in vitro* actions on cells particularly important for the development of allergic disorders. Therefore, the well-characterized eosinophil cell line EoL-1 and RBL-2H3 mast cells were used to mimic responses activated on ocular allergic reactions.

#### Proliferation and survival

**Figure 5:**
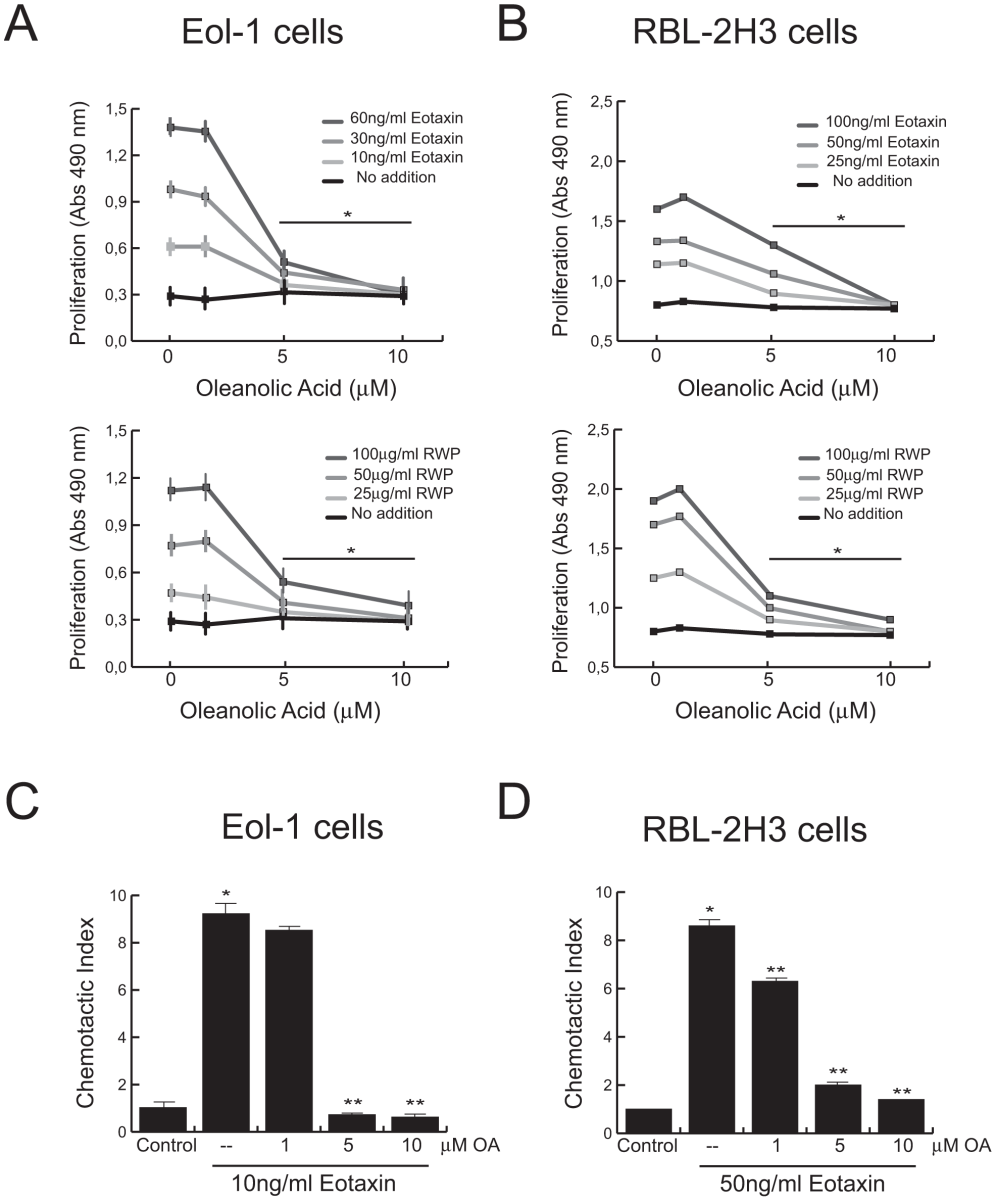
OA abrogated *in vitro* biological functions on EoL-1 and RBL-2H3 cells. EoL-1 (A,C) and RBL-2H3 (B,D) cells were incubated with the indicated stimuli in presence or absence of different concentrations of OA. (A,B) Cell proliferation was assayed 24 h after stimulation. (C,D) Cell migration was measured as described in Materials and Methods. Bars represent means ± SD. (*P<0.001 vs unstimulated cells; **P<0.001 vs stimuli without triterpene; n = 3).

EoL-1 eosinophils and RBL-2H3 mast cells were treated with different concentrations of RWP or eotaxin in presence or absence of either 5 or 10 μM of OA for 24 h. As shown in Figure 5A and B, the presence of OA significantly reduced, the proliferative response induced by the agonists, in a dose-dependent manner. The presence of OA had no significant influence on the viability of either resting or activated cells.

#### Chemotactic migration

The capacity of OA to modulate EoL-1 eosinophils and RBL-2H3 mast cells migration was assessed using Transwell chambers. As shown in Fig. 5 C and D, cell pretreatment with OA reduced in a dose-dependent manner the chemotactic activity of eotaxin in both cell lines.

**Figure 6:**
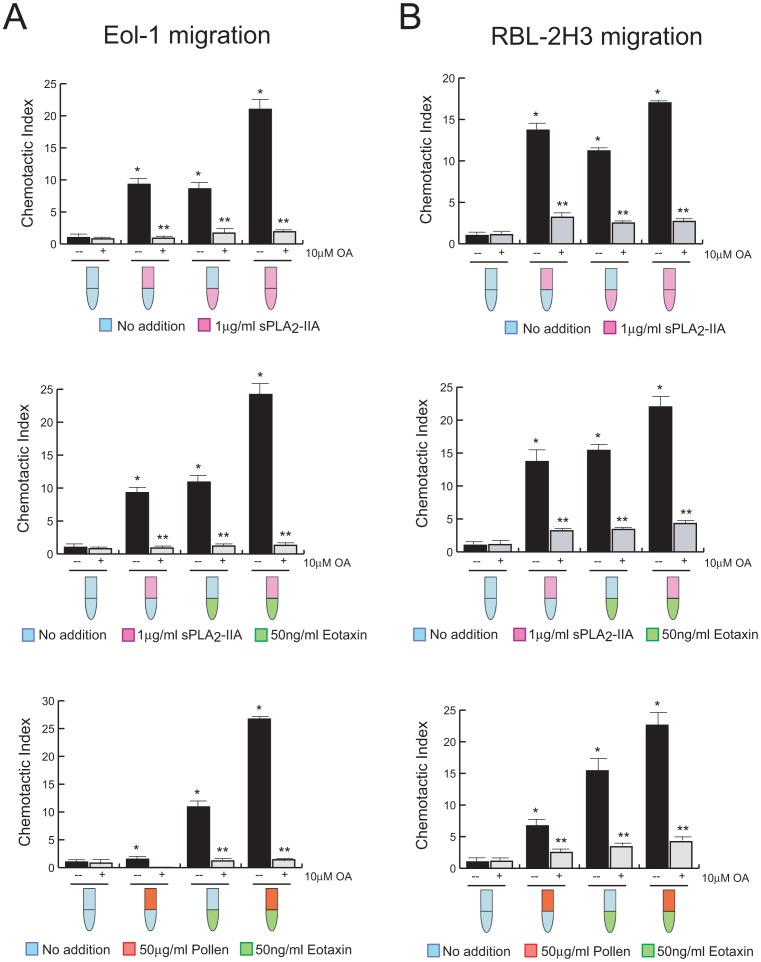
OA inhibits cell migration. EoL-1 (A) and RBL-2H3 (B) cells treated, or not, with 10 μM of OA for 30 min, were allowed to migrate as described in Materials and Methods. Cell migration was assayed using eotaxin, RWP and sPLA_2_-IIA as schematically indicated by the green, red and pink color, respectively. Bars represent means ± SD. (*P<0.001 vs untreated control condition, and **P<0.001 vs stimuli without triterpene; n = 3).

We also assessed the capacity of sPLA_2_-IIA, found up-regulated in EAC mice, and RWP to elicit and/or modify chemotactic migration. As schematically colored-indicated in Figure 6, sPLA_2_-IIA added in the lower compartment of transwell chamber induced a significant increase in EoL-1 eosinophils (Fig. 6A) and RBL-2H3 mast cells (Fig. 6B) migration over controls. In addition, both sPLA_2_-IIA- and RWP-cell pretreatment potently stimulated both spontaneous and chemoattractant-induced cell migration.

Then, we investigated the effects of OA, and we observed that cell migration was potently inhibited when cells were pre-incubated with OA. Addition of OA to the lower chamber had no detectable effect on cell motility (data not shown).

#### EoL-1 eosinophils differentiation

**Figure 7:**
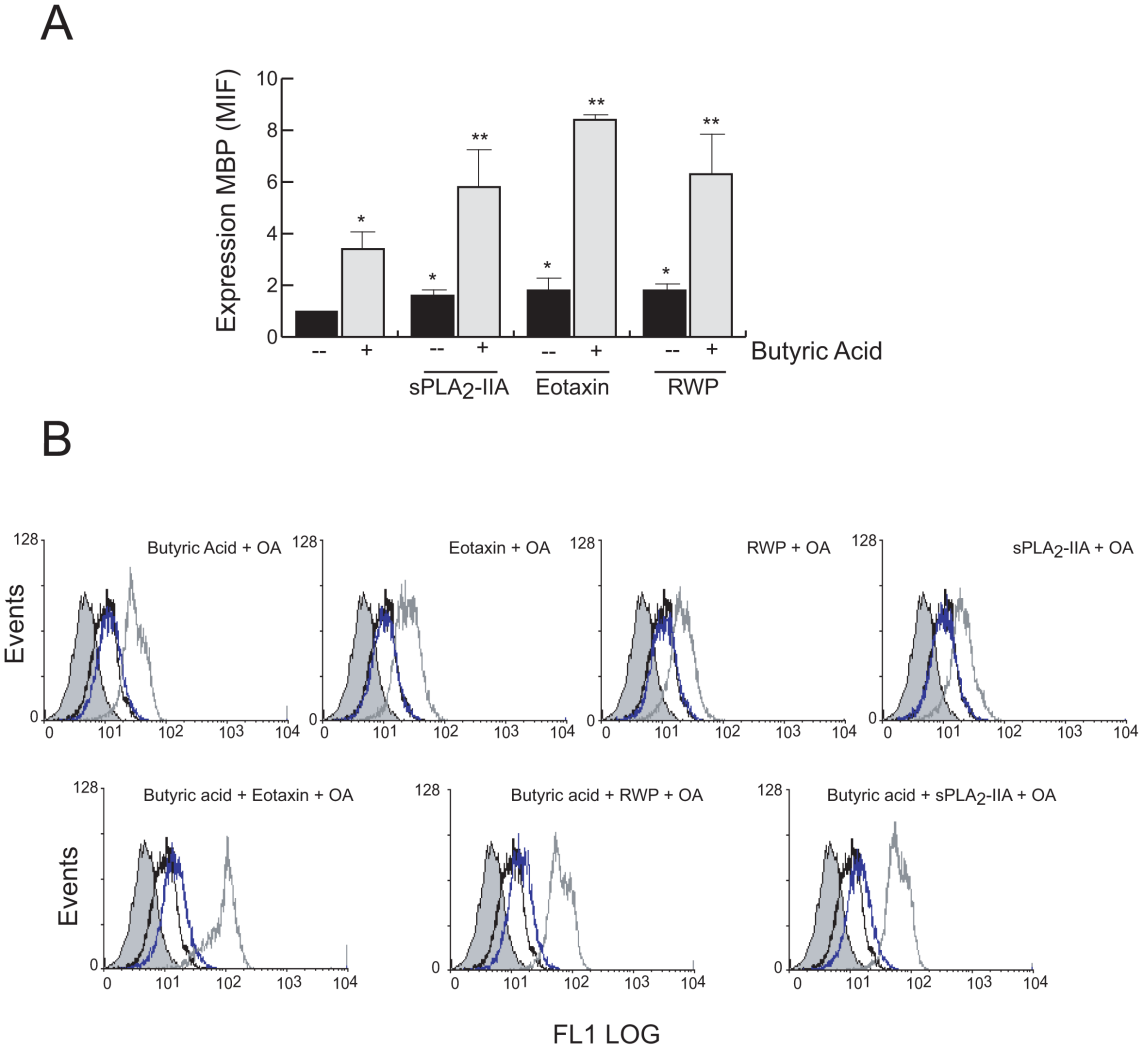
Effect OA on EoL-1 cell differentiation. EoL-1 cells were treated without and with 0.5 mM butyric acid, and 30 ng/ml of eotaxin, or 1 μg/ml sPLA_2_-IIA or 50 μg/ml RWP for 7 days, in absence (A) or presence of 5 μM OA (B). MBP expression was analyzed by Flow cytometry. In A, data are expressed as the means of MFI ± SD. *P<0.001 vs control cells, and **P<0.01 vs butyric acid-treated cells; n = 3. In B, histograms are representative of three independent experiments: untreated (open black curves), stimulated (open dark grey curves) and OA+stimulated (open blue curves). EoL-1 cells are compared with isotype controls (solid grey curves).

Differentiation of EoL-1 cells was triggered chemically with butyric acid, or with stimuli such as eotaxin, RWP and sPLA_2_-IIA, and the expression of MBP, a mature eosinophil marker, was evaluated by flow cytometry. As shown in Figure 7A all the agonists on their own promoted the upregulation of MBP at day 7 after treatment. Interestingly, when the agonists eotaxin, RWP, or sPLA_2_-IIA were added in combination with butyric acid, the differentiation process was significantly increased over butyric acid alone. As expected, the presence of OA before incubation the cells with the agonists, abrogated the differentiation process (Fig. 7B).

## Discussion

OA is a powerful anti-inflammatory agent, whose activity in ocular inflammatory disorders has never been investigated. Here, we examined the effect of OA on allergic conjunctivitis in a RWP-induced EAC model as well as its effects in vitro on eosinophils and mast cells functions. We focused on these cells since they are important mediators in the development of allergic diseases and inflammatory processes.

Intraperitoneal administration of OA, either on the sensitization day or 5 days after sensitization to an EAC model, provided strong evidence of its anti-allergic effects; while, its administration to healthy BALB/c mice did not induce signs of any pro-inflammatory response (data not sown), as found in other experimental mice models [14], [15]. The main pathophysiological changes in conjunctival allergic reactions include preferential generation of IgE antibodies in serum, as well as IgE-dependent mast cell degranulation and infiltration of eosinophils into the conjunctiva [24]. Our mouse model displayed these pathological hallmarks, and OA treatment effectively reduced the number of eosinophils and degranulated mast cells in the conjunctival tissue, and suppressed the presence of allergen-specific Igs titers in serum, showing no remarkable difference in the degree of suppression of these features between the two OA administration protocols. These findings were consistent with the decreased levels of inflammatory mediators and Th2 cytokine found in the OA-treated mice (EAC_OA10_ and EAC_OA5_).

The pathological processes of an allergic reaction are thought to be mediated by Th2-type cells which release interleukins such as IL-5 and IL-13. These cytokines are responsible for a cascade of events necessary for allergic inflammation: IgE production by B-cells, eosinophil activation and recruitment, and mast cell activation. IL-13 is a crucial mediator of hypersensivity reactions [25]–[29]. Different studies in murine models of allergen challenge have shown that IL-13 is a critical factor to induce responses that have a striking resemblance to human diseases. IL-13 regulates synthesis of IgE and coordinates the inflammatory process due to its ability to stimulate expression of adhesion molecules, chemokines and metalloproteinases, which influence the recruitment, trafficking and activation of many inflammatory cells. Meanwhile, IL-33 is another powerful inducer of allergic inflammation which has been found highly expressed in Th2-associated diseases including asthma, allergic conjunctivitis and rhinitis [30], [31]. IL-33 promotes responses which include activation and migration of Th2 lymphocytes, eosinophils, mast cells and basophiles.

Our data clearly demonstrated that the expected increased levels of IL-13, and IL-33 in the conjunctival tissue and serum of EAC mice were markedly suppressed by OA treatment. Likewise, we did see an allergen-induced increase in the release of these cytokines in splenocytes cultures after allergen challenge. Treatment with OA, both in vivo and in vitro, significantly reduced IL-13 and IL-33 cytokine production. Additionally, examination of chemokine induction revealed that the upregulated levels of eotaxin and MCP-1, in both serum and the conjunctiva of sensitized mice after allergen challenge, were substantially decreased in OA-treated mice. Similar results were found in vitro: splenocytes from the different experimental groups also showed a substantial reduction in the allergen-induced eotaxin and MCP-1 by the action of OA, along with a minimization of the proliferative response of these T-cell cultures. All of this suggests that OA was able not only to down-modulate both the systemic and local allergen-induced inflammatory response, but also to impair allergen-induced T cell responses in vivo and in vitro.

IL-13 has been shown to regulate the expression of MCP-1 and eotaxin in the respiratory and gastrointestinal mucosa, and exogenous application of IL-33 to an EAC model strongly induces local production of the chemoattractant eotaxin, and potentiates the capacity of Th2 cells to produce cytokines including IL-13 [30], [32]–[36]. At the same time, eotaxin-1 is considered as the most relevant chemokine in the pathophysiology of allergic conditions, because of its contribution to eosinophil homing or allergen-dependent chemoattraction and its regulatory function in mast cells at the ocular tissue regulating both IgE-induced degranulation and presumably maturation [30]. Therefore, the reduced presence of eosinophils and degranulated mast cells, in the conjunctiva of the OA-treated EAC mice treated, might be closely linked to the decreased expression of IL-13, IL-33 and eotaxin, all of which were clearly down-modulated by OA. Similarly, the improvement of allergic conjunctivitis in mice subjected to treatment with natural products such as curcumin or thymoquinine, an anti-oxidant and anti-inflammatory active component of *Nigella sativa*, has also been linked to its capacity to suppress Th2-driven immune responses [37], [38].

Another finding was that treatment of EAC mice with OA prevented the diminished secretion of IL-10 observed in untreated-EAC mice. In fact, the expression levels in serum and conjunctival tissue of this Treg-type cytokine involved in the Th1/Th2 homeostasis were similar in OA-treated EAC mice and in healthy control mice. It is noteworthy that in addition to its anti-inflammatory properties on human mast cells, IL-10 has recently been shown to stabilize murine mast cells and reduce their degranulation in vitro [39], [40]. Data which are in accordance with an in vivo study in a rodent model, which report that IL-10 attenuates allergic conjunctivitis by protecting from mast cell activation/degranulation, instead of affecting its numbers in the conjunctiva [40]. Similarly, our study neither revealed significant differences in the number of mast cells in the conjunctiva between untreated- and OA-treated-EAC mice (data not shown), but rather OA treatment prevented from mast cells degranulation.

In addition, we found an increased presence of sPLA_2_-IIA in serum and conjunctiva of allergic mice as compared to control healthy mice, and the administration of OA again abrogated this augmented expression. High concentrations of sPLA_2_-IIA have already been found in the tears of patients with AC both seasonal and perennial, dry eye disease, chronic blepharitis and contact lens intolerance, when compared to tears of age-matched normal controls [41]–[43]. Recently, a dry eye experimental model in mice has revealed increased expression of sPLA_2_-IIA in the goblet, as well as in epithelial cells associated with the signs and symptoms of the diseases [22]. sPLA_2_-IIA in the normal ocular surface was shown to be an innate immune barrier of the ocular surface against microbial infection, but when the ocular surface is compromised, it amplifies the inflammatory process [22], [43]. These findings might support the hypothesis that the elevated levels of sPLA_2_-IIA on conjunctiva of our EAC model might play an important role triggering ocular inflammation by affecting to the conjunctiva epithelial cells. Consequently, lowering its presence by the administration of OA should result in a diminished, or abrogated, ocular surface inflammation.

In our study we also demonstrated sPLA_2_-IIA- and allergen-specific signals in eosinophils and mast cells, which are abolished by the presence of OA. We focused on these cell-types because of its significant contribution to the development of AC due to its cytotoxic and inflammatory effects by the release of their granule proteins and the production of crucial inflammatory mediators. We showed that sPLA_2_-IIA behaves as a chemoattractant for EoL-1 eoshinophils and RBL-2H3 mast cells as potent as eotaxin, and amplifies eotaxin-induced chemotaxis similarly to what is observed with direct interaction of cells with RWP. These results are inline with previous studies demonstrating cooperative chemotactic responses between eotaxin and the cytokine IL-5 [44]. Thus, suggesting that diverse inflammatory mediators, including sPLA_2_-IIA, may act cooperatively to potentiate local chemotactic responses. In addition, the phospholipase has been shown to effectively trigger mast cell degranulation, and here we found that it enhanced EoL-1 cells to undergo morphological differentiation into mature eosinophil-like cells [45]. Interestingly, all the studied responses were abolished in cells pretreated with OA. Therefore, our data point to a critical role for sPLA_2_-IIA in the activity and migratory capability into tissues of eosinophils and mast cells, and a regulatory role for the triterpene OA in the prevention of cell recruitment and activation. However, signaling pathways involved in these events remain obscure and deserve a further and deeper investigation.

In conclusion, OA seems to be a promising candidate for the treatment of allergic eye disorders in addition to the conventional therapies. It provided an effective treatment against the allergic and immune-inflammatory responses in the EAC model. Thus, the therapeutic potential for the treatment of allergic inflammatory diseases such as allergic rhinitis and asthma should be also considered. Further studies should also be directed to explore its activity when administered topically.
